# Identifying effective characteristics of behavioral weight management interventions for people with serious mental illness: A systematic review with a qualitative comparative analysis

**DOI:** 10.1111/obr.13355

**Published:** 2021-10-20

**Authors:** Charlotte Lee, Carmen Piernas, Cristina Stewart, Moscho Michalopoulou, Anisa Hajzadeh, Rhiannon Edwards, Paul Aveyard, Felicity Waite

**Affiliations:** ^1^ Nuffield Department of Primary Care Health Sciences University of Oxford Oxford Oxfordshire UK; ^2^ Bassetlaw Hospital Doncaster and Bassetlaw Teaching Hospitals NHS Foundation Trust Worksop Nottinghamshire UK; ^3^ Department of Psychiatry University of Oxford, Warneford Hospital Oxford Oxfordshire UK; ^4^ Oxford Health NHS Foundation Trust Oxford Oxfordshire UK

**Keywords:** bipolar, schizophrenia, treatment, weight

## Abstract

People with serious mental illness (SMI) have identified barriers to engaging in behavioral weight management interventions (BWMIs). We assessed whether BWMIs that addressed these barriers were more effective. First, we systematically reviewed qualitative literature and used a thematic analysis to identify the characteristics of BWMIs that promote engagement for adults with SMI. Second, we systematically reviewed randomized controlled trials (RCTs) of BWMIs in adults with SMI. Data on the characteristics that promoted engagement and weight outcomes were extracted. We then used a crisp‐set qualitative comparative analysis (CsQCA) to identify which characteristics were associated with weight loss. For the qualitative review, 20 studies in 515 people with SMI were analyzed and nine characteristics were reported to promote engagement in BWMIs. For the systematic review, 34 RCTs testing 36 interventions in 4305 participants were included. The active interventions resulted in more weight loss (mean = −4.37 to +1 kg at 6 weeks to 18 months follow‐up) compared with controls (−1.64 to +3.08 kg). The CsQCA showed BWMIs that offered regular contact, tools to support enactment, and tailored materials were associated with effectiveness. As these are all supplementary strategies, it may be possible to augment BWMIs available for the general population to engage people with SMI.

## 1 INTRODUCTION

The global prevalence of overweight (body mass index [BMI] 25–29.9 kg/m^2^) and obesity (BMI > 30 kg/m^2^) is increasing and its adverse effects on health are well‐documented.^1^, ^2^ Overweight and obesity are 2 to 3 times more common in people with serious mental illness (SMI) defined as psychotic disorders like schizophrenia and bipolar disorder.^3^ These disorders are often long‐term mental health diagnoses marked by hearing, seeing, or believing things that are not real.^4^ Antipsychotic medications are sometimes used to manage the symptoms of SMI but contribute to excess weight through increased appetite and metabolic changes.^5^ The risk of excess weight and metabolic disturbance appears higher with second‐generation drugs, particularly olanzapine and clozapine.^6^ Poor diet and physical inactivity also cause excess weight and these are more common in people with SMI compared with the general population.^7^ The higher prevalence of overweight and obesity contributes to a higher incidence of cardiovascular disease (CVD) in people with SMI, which is the main factor that reduces their life expectancy by 15 to 20 years.^8^ Hence, addressing overweight and obesity in people with SMI is of utmost importance.

In the general population, randomized controlled trials (RCTs) of behavioral weight management interventions (BWMIs) have supported people to follow an energy‐restricted diet and increase physical activity. These trials have produced greater weight loss than without support,^9^, ^10^ and have shown to reverse type 2 diabetes, lower hypertension, and improve lipid profiles.^11^ Accordingly, national guidelines in the United States and United Kingdom suggest offering BWMIs to achieve weight loss for anyone with overweight or obesity.^12^, ^13^ These BWMIs are the mainstay treatment for overweight and obesity in many high‐income countries and are provided as part of healthcare services.^14^ However, people with SMI have reported barriers to engaging with standard BWMIs.^15^ These include anxiety in social situations arising from fear of harm from others (i.e., persecutory beliefs) or hearing threatening or critical voices (i.e., auditory hallucinations).^16^ Distressing beliefs about oneself related to low self‐esteem can undermine persistence with weight loss attempts.^16^ People with SMI can also experience difficulties in concentration and motivation.^17^ Such barriers have led researchers to develop and test BWMIs that are bespoke for people with SMI.

Previous systematic reviews of these bespoke BWMIs show evidence that, overall, they can be effective but with heterogeneity. For example, Speyer et al. reported BWMIs were effective in reducing weight compared with treatment as usual (TAU) but with moderate heterogeneity: pooled effect = −2.20 kg, 95% CI −3.01 to −1.42 kg, *p* < 0.001, *I* ^2^ = 35.1%.^18^ Differences across the intervention characteristics may explain these results. Furthermore, while bespoke BWMIs for people with SMI can be effective, they are rarely provided as part of routine healthcare provision. Therefore, we assess how BWMIs have tailored support to overcome the barriers to engagement people with SMI experience, and assess how differences in these intervention characteristics explain difference in weight loss. Our aim is to inform researchers on how standard BWMIs may be adapted to better serve people with SMI. Specifically, we

- systematically review qualitative studies to identify which characteristics of BWMIs promote engagement for people with SMI using a thematic analysis;
- systematically review RCTs to identify the characteristics of behavioral weight management interventions associated with weight loss using a crisp‐set qualitative comparative analysis (CsQCA).

## 2 METHODS

A protocol was registered in advance and is available in PROSPERO (CRD42020189897). Reporting followed the Preferred Reporting Items for Systematic Reviews and Meta‐Analyses (PRISMA) statement.^19^

### 2.1 Patient and public involvement

We consulted 12 members of the UK public with lived‐experience of SMI. We aimed to ensure the research question was relevant and to use their feedback to inform data interpretation. Ethical approval was obtained from the University of Oxford Medical Sciences Interdivisional Research Ethics Committee (R68892/RE001).

The patient and public involvement (PPI) contributors were recruited via local networks within the University of Oxford and The McPin Foundation. We obtained informed consent over the telephone. We then conducted individual telephone interviews or online focus groups between August 14 and October 9, 2020. All discussions were guided by a semistructured topic guide (Appendix A).

In total, we conducted five telephone interviews and two focus groups—one of four contributors, one of three contributors. Each consultation lasted 2 h with scheduled breaks every 30 min. All consultations were facilitated, audio‐recorded, and transcribed by the first author. Next, we used a thematic synthesis of the data guided by the Enhancing Transparency in Reporting the Synthesis of Qualitative Research (ENTREQ) guidelines.^20^ Thematic synthesis aims to accumulate and summarize descriptive patterns in data rather than transform it for new theories.^21^, ^22^, ^23^ Using this method, the first author coded line‐by‐line each transcript to produce an initial coding frame of intervention characteristics that promote engagement in BWMIs. This coding frame was developed by the lead author and reviewed by members of the research team. The coding frame was then augmented with our systematic review of qualitative studies (Section 2.2  below).

### 2.2 Systematic review of qualitative studies

#### 2.2.1 Eligibility criteria and search strategy

We aimed to review qualitative studies to identify which characteristics of BWMIs promote engagement for people with SMI.

We included peer‐reviewed qualitative studies. This included studies reporting any qualitative element of an intervention and RCTs that reported the results of nested qualitative studies. We searched MEDLINE (OvidSP) (1946 to present) from database inception to September 23, 2020, using text word terms (Appendix B).

We also searched for studies that reported qualitative enquiries that aimed to assess the response of people with SMI to eating healthy outside of an intervention. In addition, we searched reference lists of all included studies. We excluded studies that solely focused on children and people without a nonpsychotic mental illness (i.e., eating or neurodevelopmental disorders or stakeholders only). We also excluded entirely quantitative studies. No restrictions were set on the date of publication, language, or care setting.

#### 2.2.2 Data synthesis and analysis

We used a thematic synthesis of the data guided by the ENTREQ guidelines.^20^ Using this method, data analysis proceeded as follows. First, we used the coding frame developed from the PPI consultations to inform our subsequent data interpretation. Next, the lead author coded line‐by‐line the result and discussion sections of the included studies to augment the coding frame with new themes. Codes were then grouped into broader categories of shared meaning. Categories were then summarized to produce top‐level analytical themes of intervention characteristics that promote engagement in BWMIs for people with SMI. A second reviewer, who was closely involved with both the PPI consultations and the systematic review of qualitative studies, verified the finalized groupings of analytical themes. Finally, all data were presented to our PPI contributors for validation. Data were coded and managed using NVivo 11 software.^24^ Selected quotations are presented in the results section and names have been anonymized.

### 2.3 Systematic review of randomized trials

We conducted a systematic review of RCTs of BWMIs to identify which characteristics are associated with clinical effectiveness. The systematic search started on June 11, 2020, after the protocol was approved and registered in PROSPERO, though data extraction began once the above intervention characteristics were finalized on October 28, 2020. Methods for the searching, screening, data extraction, and quality assessment of studies followed the Cochrane handbook guidelines.^25^

#### 2.3.1 Eligibility criteria

Articles included met the following criteria:

- **Population**: Adults (aged ≥18 years, no upper limit); with SMI defined by a primary diagnosis of psychosis (i.e., schizophrenia, schizophreniform disorder, schizoaffective disorder, delusional disorder, brief reactive psychosis, psychosis not otherwise specified) or bipolar disorder; and who had overweight (BMI 25–29.9 kg/m^2^) or obesity (>30 kg/m^2^, no upper limit). Studies on people with a diagnosis of a nonpsychotic mental illness were excluded. There was no restriction on medication use.
- **Intervention**: Individual or cluster RCTs of any behavioral (i.e., nonpharmacological or bariatric) intervention that aimed to support weight management (i.e., defined as weight maintenance or weight loss) through diet alone or diet and physical activity. To refine the scope of this review, we excluded studies that focused solely on physical activity. No restrictions were set based on intervention characteristics or duration.
- **Comparison**: Any comparison conditions including other BWMIs or TAU. For studies including another BWMI as a comparison, we isolated the intervention characteristics not included in the control group (i.e., only included in the active intervention group[s]) and recorded these in the data extraction form.
- **Outcomes**: Mean weight change (kg), BMI (kg/m^2^), or percentage weight change (kg). When measured on multiple occasions, only data at the first follow‐up postintervention was extracted.

#### 2.3.2 Search strategy

The search strategy was co‐developed by the research team with a specialist health science librarian at the University of Oxford. The following databases were searched from database inception until June 11, 2020, using medical subject headings, or similar when possible, or text word terms: Medline, EMBASE (OvidSP) (1974 to present), PsychINFO (OvidSP) (1806 to present), and CINAHL (EBSCOHost) (1982 to present). We also searched reference lists of included studies and previous systematic reviews.^18^, ^26^, ^27^, ^28^, ^29^ No year or language limits were set. The Medline search strategy is provided in Appendix C.

#### 2.3.3 Study selection and data extraction

All studies identified were imported into Covidence for screening.^30^ After duplicates were removed, titles and abstracts were double‐screened for eligibility. Discrepancies regarding study inclusion were resolved through discussion. Data were double extracted by five researchers using a piloted form. The data extracted included: participant characteristics (i.e., age, sex, and SMI diagnosis); characteristics of the intervention identified from the qualitative review, as well as characteristics of the control group; length of follow‐up; and weight outcomes. Authors were contacted for further information where necessary.

#### 2.3.4 Risk of bias assessment

Risk of bias (RoB) assessments were conducted in duplicate using the Cochrane risk of bias tool.^25^ The following bias domains were assessed as low, high, or unclear risk: allocation sequence generation, allocation concealment, blinding of outcome assessors, incomplete outcome data, selective outcome reporting, and other bias. It is not possible to blind participants or study personnel to allocation in behavioral intervention trials so we omitted this domain.

#### 2.3.5 Data synthesis and analysis

We did not perform a meta‐analysis due to anticipated heterogeneity across intervention design and implementation. Instead, we conducted a narrative synthesis of the data guided by the Synthesis Without Meta‐analysis (SWiM) reporting guidelines.^31^ Using this approach, we grouped studies by end‐of‐intervention duration (i.e., ≤6 or 7–12 months). The results were augmented with an exploratory crisp‐set qualitative comparative analysis (CsQCA).^32^, ^33^ This method aims to establish causal relationships through systematic comparisons. Using this method, data analysis preceded in the following stages. The first stage relied on our systematic review of qualitative studies which identified characteristics (i.e., conceptual categories) from the literature. These characteristics formed the conditions that were examined in the CsQCA. In the next stage, each intervention arm (i.e., case) identified from systematic review of randomized trials was coded for either the presence (=1) or absence (=0) of the characteristic. Interventions were also coded as effective (=1) or not (=0) depending on whether there was a statistically significant (*p ≤* 0.05) difference in weight at end‐of‐intervention follow‐up. Next, a raw data matrix and truth table were created to code these characteristics and outcomes, which was used in the CsQCA. In interpreting the results of the CsQCA, two concepts were key: consistency and coverage. Consistency refers to the percentage of characteristics that were present in interventions that resulted in a statistically significant between‐group difference in weight at follow‐up. Consistency is the proportion of times an intervention is effective when a particular characteristic is present. Characteristics that contribute to effectiveness would lead to high consistency (possible range from 0 to 1, with high consistency indicated by ≥0.75). Coverage refers to the proportion of effective interventions in which a particular characteristic is present. Given there are several plausibly effective characteristics, low coverage does not indicate lack of a valid association between cause and effect, only that it is less commonly present in effective interventions.

## 3 RESULTS

### 3.1 Patient and public involvement

Overall, people with a lived experience of SMI recognized the need to manage their weight and were positive about the opportunity for more support. The results of the interviews and focus groups are presented in the coding frame in Appendix D. The coding frame was further developed using the results of the systematic review of qualitative studies and the final (combined) themes are presented below.

### 3.2 Systematic review of qualitative studies

**FIGURE 1:**
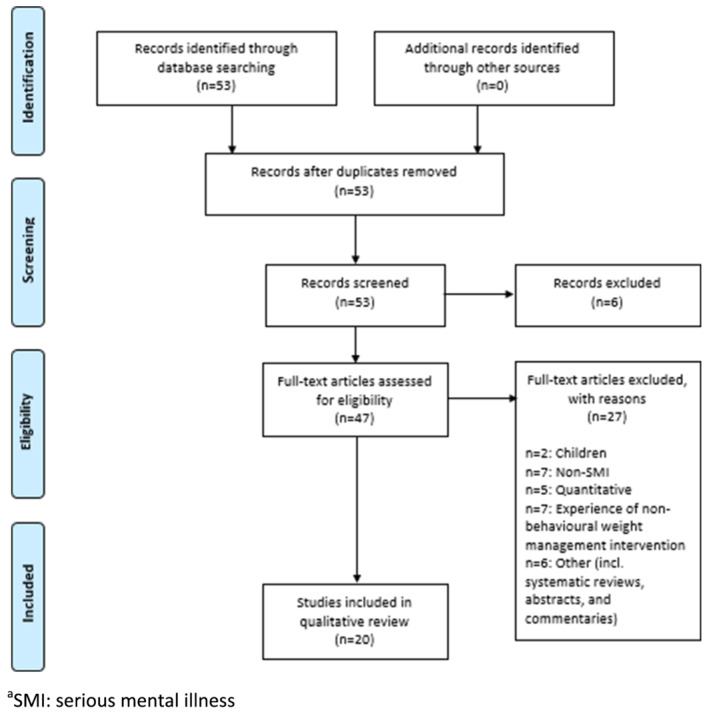
PRISMA flow diagram for the systematic review of qualitative studies

As shown in Figure 1, 53 studies were retrieved for full text search and 20 studies were included representing 515 individual participants.^34^, ^35^, ^36^, ^37^, ^38^, ^39^, ^40^, ^41^, ^42^, ^43^, ^44^, ^45^, ^46^, ^47^, ^48^, ^49^, ^50^, ^51^, ^52^, ^53^ Of these studies, 15 studies specified age and the median was 47 years (range: 38–55).^35^, ^37^, ^39^, ^40^, ^41^, ^42^, ^43^, ^44^, ^45^, ^46^, ^47^, ^49^, ^50^, ^51^, ^52^ Thirteen studies specified sex and 41% were male.^35^, ^37^, ^39^, ^40^, ^41^, ^42^, ^43^, ^44^, ^45^, ^46^, ^47^, ^48^, ^49^, ^50^, ^51^, ^52^ In the 11 studies that reported ethnicity, on average 53% of participants were white.^35^, ^37^, ^39^, ^42^, ^43^, ^44^, ^45^, ^47^, ^49^, ^51^, ^52^

Fourteen were conducted in the United States^34^, ^35^, ^36^, ^37^, ^38^, ^39^, ^42^, ^43^, ^44^, ^45^, ^46^, ^47^, ^52^, ^53^; three in the United Kingdom^41^, ^48^, ^51^; and one each in Australia,^40^ New Zealand,^49^ and South India.^50^ Eleven of the 20 studies were conducted with people living in the community,^34^, ^35^, ^37^, ^41^, ^44^, ^45^, ^46^, ^48^, ^50^, ^51^ and eight were facilitated by research staff.^37^, ^38^, ^41^, ^43^, ^45^, ^47^, ^49^, ^50^

Three reported participants' response to proposed intervention characteristics, prior to implementation, that were qualitatively assessed^35^, ^38^, ^48^; 12 related to participants' experiences of an intervention as part of a trial^34^, ^36^, ^37^, ^39^, ^40^, ^43^, ^44^, ^45^, ^47^, ^51^, ^52^, ^53^; one reported on the perspectives of those who declined to participate in a trial^41^; and the remaining four reported participants' views on factors relating to weight gain and following a healthy lifestyle.^42^, ^46^, ^49^, ^50^ A summary of participant‐ and study‐level characteristics is provided in Table 1 (see also Appendix E).

**TABLE 1:** Summary of participant‐ and study‐level characteristics for the systematic review of qualitative studies

	Number of studies, *n* (%)	Citations
**Study design**		
Qualitative	20 (100%)	^34^, ^35^, ^36^, ^37^, ^38^, ^39^, ^40^, ^41^, ^42^, ^43^, ^44^, ^45^, ^46^, ^47^, ^48^, ^49^, ^50^, ^51^, ^52^, ^53^
**Participant characteristics**		
Age	16 (75%)	^35^ ^,^ ^37^ ^,^ ^39^, ^40^, ^41^, ^42^, ^43^, ^44^, ^45^, ^46^, ^47^, ^48^, ^49^, ^50^, ^51^, ^52^
Years, median (range)	47 (38–55 years)	
Unclear	1 (5%)	^38^
Not reported	4 (20%)	^34^ ^,^ ^36^ ^,^ ^48^ ^,^ ^53^
Sex	13 (70%)	^34^, ^35^, ^36^, ^37^ ^,^ ^40^ ^,^ ^42^, ^43^, ^44^, ^45^ ^,^ ^47^ ^,^ ^49^ ^,^ ^51^, ^52^, ^53^
Male, %	41%	
Unclear	3 (15%)	^38^ ^,^ ^39^ ^,^ ^41^
Not reported	4 (20%)	^36^ ^,^ ^46^ ^,^ ^48^ ^,^ ^50^
Ethnicity	11 (55%)	^35^ ^,^ ^37^ ^,^ ^39^ ^,^ ^42^, ^43^, ^44^, ^45^ ^,^ ^47^ ^,^ ^49^ ^,^ ^51^ ^,^ ^52^
White, %	53%	
Unclear	1 (5%)	^38^
Not reported	8 (40%)	^34^ ^,^ ^36^ ^,^ ^40^ ^,^ ^41^ ^,^ ^46^ ^,^ ^48^ ^,^ ^50^ ^,^ ^53^
**Study country**		
USA	14 (70%)	^34^, ^35^, ^36^, ^37^, ^38^, ^39^ ^,^ ^42^, ^43^, ^44^, ^45^, ^46^, ^47^ ^,^ ^52^ ^,^ ^53^
Australia	1 (5%)	^40^
New Zealand	1 (5%)	^49^
South India	1 (5%)	^50^
UK	3 (15%)	^41^ ^,^ ^48^ ^,^ ^51^
Unclear	0 (0%)	None
Not reported	0 (0%)	None
**Study characteristics**		
**Care‐setting**		
Outpatients/community mental health teams	11 (55%)	^34^, ^35^, ^36^, ^37^ ^,^ ^41^ ^,^ ^44^, ^45^, ^46^ ^,^ ^48^ ^,^ ^50^ ^,^ ^51^
Inpatients	0 (0%)	None
Both	0 (0%)	None
Supportive housing	3 (15%)	^38^ ^,^ ^42^ ^,^ ^47^
Other	1 (5%)	^49^
Unclear	0 (0%)	None
Not reported	5 (25%)	^39^ ^,^ ^40^ ^,^ ^43^ ^,^ ^52^ ^,^ ^53^
**Facilitator**		
Mental health professionals (e.g., clinical psychologist)	1 (5%)	^40^
Other health professional (e.g., nurse)	1 (5%)	^53^
Dietitians	0	None
Research staff	8 (40%)	^37^ ^,^ ^38^ ^,^ ^41^ ^,^ ^43^ ^,^ ^45^ ^,^ ^47^ ^,^ ^49^ ^,^ ^50^
Mix facilitators	0	None
Other	2 (10%)	^42^ ^,^ ^44^
Unclear	2 (10%)	^34^ ^,^ ^39^
Not reported	6 (30%)	^35^ ^,^ ^36^ ^,^ ^46^ ^,^ ^48^ ^,^ ^51^ ^,^ ^52^
**Delivery format**		
One‐to‐one	15 (75%)	^35^ ^,^ ^37^ ^,^ ^39^ ^,^ ^40^ ^,^ ^41^ ^,^ ^43^, ^44^, ^45^, ^46^, ^47^ ^,^ ^49^ ^,^ ^50^, ^51^, ^52^, ^53^
Focus group	4 (20%)	^34^ ^,^ ^38^ ^,^ ^42^ ^,^ ^48^
Both	0	None
Unclear	0	None
Not reported	1 (5%)	^36^

The thematic analysis identified nine characteristics that promoted engagement for people with SMI BWMIs. These are outlined below:

#### 3.2.1 Education on the specific contributors to weight gain for people with SMI

Participants understood what constitutes a healthy diet. They were less clear on how the effects of some antipsychotic medications would affect their ability to manage weight. Interventions that discussed this improved some participants' knowledge and confidence, and subsequent involvement in the study.

Definitely. I think if I'd had known about [the side‐effects of the antipsychotic medications] I would have been a bit more prepared to spot [the weight gain] and maybe done something, you know? PPI, female

#### 3.2.2 Emphasis on successes and achievements

Lapses in a diet program and/or continued weight gain contributed to low self‐esteem. In turn, this undermined motivation and self‐efficacy to continue with the BWMI. For this reason, participants valued interventions that emphasized their successes and praised achievements rather than perceived failings.

My family is starting to notice that I'm losing weight. I like the positive comments … I feel like I've got more energy and more motivation to do stuff.^45^

#### 3.2.3 Knowledgeable facilitator

The symptoms of SMI, along with societal stigma about these symptoms, can lead people to withdraw from situations like a BWMI. Participants emphasized that it was important the person providing the intervention understood the nature of SMI and conveyed empathy and respect. Ideally, participants wanted support from a mental health professional.

A non‐judgemental and sympathetic person who is not going to shame [me]. PPI, male

#### 3.2.4 Peer support

Similarly, participants valued opportunities to connect with other participants in the BWMI (e.g., attending an exercise or cookery class together). It was noted when this was absent.

One of the most important things was being part of the group; I enjoyed being with people and not having to do things on my own.^40^

[Being] in a group, we have the support, safety and strength from your friends rather than being frightened or anxious with strangers.^50^

#### 3.2.5 Interim booster support

People with SMI reported difficulties initiating weight loss tasks owing to fluctuating symptoms, medication side effects, and varying motivation. Participants valued proactive support between sessions (e.g., telephone calls) to help translate intentions into action. It also provided an added opportunity to foster therapeutic rapport with the person who was facilitating the intervention, and reduced feelings of isolation.

Call reminders as people forget about appointments. Text or phone OK.^48^

… having somebody to report to … it makes me feel good to say “Shirley, I went to the gym three times this week,” and she's proud of me because I did it. That's important to me, having somebody to say I did it ….^43^

#### 3.2.6 Supporting tools

Participants valued tools (e.g., intervention handbooks, pedometers, cookery books) that could help initiate a weight loss activity.

The introduction of supporting tools … supported the messages provided to participants about the benefits of participation, improved internal motivation, and supported engagement and attendance.^48^

#### 3.2.7 Tailored materials

Tailored content (e.g., materials written in plain and simple language) and structure (e.g., shorter or repeated sessions) could make it easier for participants to engage in the intervention while experiencing symptoms of SMI (e.g., psychotic experience or anxiety).

“Duration of a session should not exceed two hours. Long sessions could cause anxiety [and] be difficult for people on [antipsychotic depot] injections” and “regular breaks are important for concentration”.^48^

#### 3.2.8 Practical support

Organized logistics around session attendance (e.g., transport provision, or medical clearance for studies conducted in the United States) helped reduce fears and anxieties of traveling to unfamiliar places, and maintained attendance.

None of the 10 participants were using the local recreation center … citing feelings of isolation, high cost, and transportation difficulties.^53^

Transport [was] a problem – [I] had to catch two buses to get to the venue. [My] own mental health can get in the way of attending.^48^

Several participants received help with transportation [which] appeared to combine practical and emotional support for some participants.^34^

#### 3.2.9 Incentives

Some participants reported low socioeconomic status and living in neighborhoods with limited access to healthier food. Incentives, like free food samples and food tokens, were therefore welcomed by participants.

To have a diet is not easy. Things are very expensive. That's something that stands in my way from getting the good nutrition, from buying nutritious stuff. I don't got the income to do it.^52^

… they said … introduce a little variety … I put … half a can of green chili in my beans and there went my budget.^39^

### 3.3 Systematic review of randomized trials

#### 3.3.1 Study selection

**FIGURE 2:**
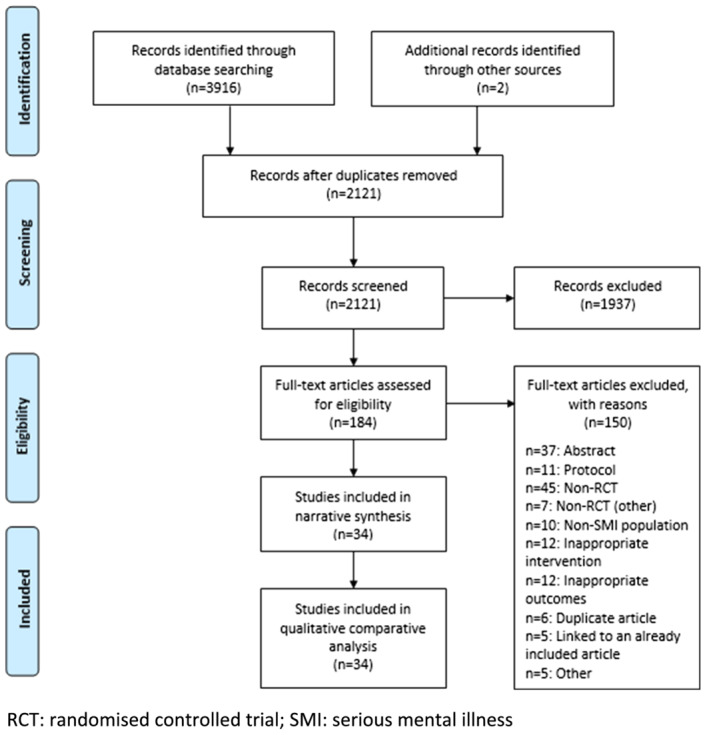
PRISMA flow diagram for the systematic review of randomized trials

As shown in Figure 2, the title and abstracts of 2121 unique studies were screened. Full‐text studies were assessed for 184 records. In total, 34 studies met the inclusion criteria and were included for the CsQCA.^54^, ^55^, ^56^, ^57^, ^58^, ^59^, ^60^, ^61^, ^62^, ^63^, ^64^, ^65^, ^66^, ^67^, ^68^, ^69^, ^70^, ^71^, ^72^, ^73^, ^74^, ^75^, ^76^, ^77^, ^78^, ^79^, ^80^, ^81^, ^82^, ^83^, ^84^, ^85^, ^86^, ^87^ Two studies were included twice in the CsQCA because they each contributed to two intervention arms.^71^, ^81^

#### 3.3.2 Participants characteristics

All studies were individually randomized trials and represented 4,305 individual participants. In the 16 studies that specified age, the median age was 44 years (range: 26–52)^54^, ^56^, ^57^, ^61^, ^66^, ^67^, ^72^, ^73^, ^74^, ^75^, ^78^, ^79^, ^80^, ^85^, ^86^, ^87^ and one study reported a median age of 57.^59^ All studies reported sex and 43% of participants were male.^54^, ^55^, ^56^, ^57^, ^58^, ^59^, ^60^, ^61^, ^62^, ^63^, ^64^, ^65^, ^66^, ^67^, ^68^, ^69^, ^70^, ^71^, ^72^, ^73^, ^74^, ^75^, ^76^, ^77^, ^78^, ^79^, ^80^, ^81^, ^82^, ^83^, ^84^, ^85^, ^86^, ^87^ In the 18 studies that reported ethnicity, on average 60% of participants were white.^56^, ^59^, ^60^, ^63^, ^66^, ^67^, ^68^, ^69^, ^71^, ^72^, ^73^, ^74^, ^75^, ^77^, ^78^, ^80^, ^83^, ^87^ In the 27 studies that specified participants' diagnoses, 67% of participants had schizophrenia spectrum disorder.^55^, ^56^, ^57^, ^58^, ^60^, ^61^, ^62^, ^63^, ^65^, ^66^, ^67^, ^68^, ^69^, ^70^, ^72^, ^73^, ^74^, ^75^, ^76^, ^77^, ^78^, ^79^, ^80^, ^81^, ^85^, ^86^, ^87^

Fourteen of the 34 studies were conducted in the United States^59^, ^60^, ^63^, ^67^, ^69^, ^71^, ^72^, ^73^, ^74^, ^75^, ^77^, ^80^, ^83^, ^87^; four in Spain^54^, ^56^, ^62^, ^85^; two each in Australia^55^, ^68^; Italy,^61^, ^64^ Switzerland,^57^, ^86^ and the United Kingdom^78^, ^82^; and one each in Brazil,^66^ Croatia,^65^ Germany,^84^ Japan,^81^ Korea,^58^ Sweden,^76^ Taiwan,^70^ and the Netherlands.^79^

#### 3.3.3 Study characteristics

Overall, 22 studies were conducted with people living in the community,^56^, ^58^, ^59^, ^60^, ^61^, ^63^, ^66^, ^68^, ^69^, ^71^, ^72^, ^73^, ^74^, ^75^, ^77^, ^78^, ^81^, ^82^, ^83^, ^85^, ^86^, ^87^ three were conducted with inpatients,^65^, ^70^, ^84^ two included both outpatients and inpatients,^54^, ^55^ one was conducted with patients and staff in supported housing facilities,^76^ and three studies did not report this.^62^, ^64^, ^67^ Two studies aimed to support weight maintenance after initiating antipsychotic medication,^54^, ^55^ the other 32 studies aimed to support weight loss.

The duration of the interventions ranged from 6 weeks to 18 months (median 18 weeks) and were delivered by mental health professionals,^54^, ^56^, ^57^, ^66^, ^69^, ^78^, ^79^, ^81^, ^85^ research staff,^59^, ^67^, ^68^, ^74^, ^82^, ^87^ and dietitians.^55^, ^84^ The interventions used one or more of: education or instruction, behavioral therapy, and motivational interviewing. The educational components focused on the constituents and benefits of a healthy diet. The instructional components typically promoted energy‐restriction by decreasing portion sizes and free‐sugar soft drinks, swapping to healthier alternatives and increasing physical activity. Four of these interventions encouraged participants to reduce their calorie intake by around 500 kcal per day.^64^, ^65^, ^70^, ^83^ The behavioral therapy comprised goal‐setting and problem‐solving strategies to promote control over calorie intake and cues to eat.

The comparison group were offered TAU (i.e., no weight loss support) in all but three studies.^59^, ^72^, ^73^ In one study, the control group received a monthly newsletter about healthy eating.^59^ In the other two studies, the control group were offered a free membership to the same local fitness club plus educational materials without access to a health mentor.^72^, ^73^

On average, BWMIs included a mean of three of the nine intervention characteristics identified in the qualitative thematic analysis. The BWMIs that were specific to people with SMI included, on average, six of the characteristics. Across all studies, the most common characteristic was an intervention that was facilitated by a mental health professional, which was included in 25 interventions representing 23 studies.^54^, ^56^, ^57^, ^59^, ^60^, ^62^, ^66^, ^67^, ^68^, ^69^, ^71^, ^72^, ^73^, ^74^, ^75^, ^76^, ^78^, ^79^, ^81^, ^84^, ^85^, ^86^, ^87^ The least common characteristics were interim booster support^59^, ^60^, ^71^, ^75^, ^78^, ^83^ and practical support^68^, ^69^, ^72^, ^73^, ^78^, ^86^ which were both included in only six interventions. In all cases, the interim booster support involved telephone calls, or other unspecified support, from the person facilitating the intervention.^59^, ^60^, ^71^, ^75^, ^78^, ^83^ In five interventions, this was a weekly telephone call,^59^, ^60^, ^71^, ^75^, ^83^ and fortnightly in one intervention.^78^ The nature of the call was not specified. The mean weight change in the intervention groups lay between −4.37 to +1 kg at 6 weeks to 18 months follow up, compared with −1.64 to +3.08 kg in the control group. A summary of participant‐ and study‐level characteristics is provided in Table 2 (see also Appendices F and G).

**TABLE 2:** Summary of participant‐ and study‐level characteristics for the systematic review of randomized trials

	Number of studies, *n* (%)	Citations
**Study design**		
RCT	34 (100%)	^54^, ^55^, ^56^, ^57^, ^58^, ^59^, ^60^, ^61^, ^62^, ^63^, ^64^, ^65^, ^66^, ^67^, ^68^, ^69^, ^70^, ^71^, ^72^, ^73^, ^74^, ^75^, ^76^, ^77^, ^78^, ^79^, ^80^, ^81^, ^82^, ^83^, ^84^, ^85^, ^86^, ^87^
Unclear	0 (0%)	None
Not reported	0 (0%)	None
**Participant characteristics**		
Age	16 (47%)	^54^ ^,^ ^56^ ^,^ ^57^ ^,^ ^59^ ^,^ ^61^ ^,^ ^67^ ^,^ ^72^, ^73^, ^74^, ^75^ ^,^ ^78^, ^79^, ^80^ ^,^ ^85^, ^86^, ^87^
Years, median (range)	44 (26–52 years)	
Unclear	0 (0%)	None
Not Reported	18 (53%)	^55^ ^,^ ^58^ ^,^ ^60^ ^,^ ^62^, ^63^, ^64^, ^65^, ^66^ ^,^ ^68^, ^69^, ^70^, ^71^ ^,^ ^76^ ^,^ ^77^ ^,^ ^81^, ^82^, ^83^, ^84^
Sex	34 (100%)	^54^, ^55^, ^56^, ^57^, ^58^, ^59^, ^60^, ^61^, ^62^, ^63^, ^64^, ^65^, ^66^, ^67^, ^68^, ^69^, ^70^, ^71^, ^72^, ^73^, ^74^, ^75^, ^76^, ^77^, ^78^, ^79^, ^80^, ^81^, ^82^, ^83^, ^84^, ^85^, ^86^, ^87^
Male, %	43%	
Unclear	0 (0%)	None
Not reported	0 (0%)	None
Ethnicity	18 (53%)	^56^ ^,^ ^59^ ^,^ ^60^ ^,^ ^63^ ^,^ ^66^, ^67^, ^68^, ^69^ ^,^ ^71^, ^72^, ^73^, ^74^, ^75^ ^,^ ^77^ ^,^ ^78^ ^,^ ^80^ ^,^ ^83^ ^,^ ^87^
White, %	60%	
Unclear	0 (0%)	None
Not reported	16 (47%)	^54^ ^,^ ^55^ ^,^ ^57^ ^,^ ^58^ ^,^ ^61^ ^,^ ^62^ ^,^ ^64^ ^,^ ^65^ ^,^ ^70^ ^,^ ^72^ ^,^ ^79^ ^,^ ^81^ ^,^ ^82^ ^,^ ^84^, ^85^, ^86^
Diagnoses	27 (79%)	^55^, ^56^, ^57^, ^58^ ^,^ ^60^, ^61^, ^62^, ^63^ ^,^ ^65^, ^66^, ^67^, ^68^, ^69^, ^70^ ^,^ ^72^, ^73^, ^74^, ^75^, ^76^, ^77^, ^78^, ^79^, ^80^, ^81^ ^,^ ^85^, ^86^, ^87^
Schizophrenia	24 (89%)	^55^, ^56^, ^57^, ^58^ ^,^ ^60^ ^,^ ^62^ ^,^ ^63^ ^,^ ^65^ ^,^ ^66^ ^,^ ^68^ ^,^ ^69^ ^,^ ^70^ ^,^ ^72^, ^73^, ^74^, ^75^, ^76^, ^77^, ^78^, ^79^, ^80^, ^81^ ^,^ ^85^ ^,^ ^87^
Schizoaffective disorder	10 (37%)	^55^ ^,^ ^60^, ^61^, ^62^ ^,^ ^72^, ^73^, ^74^ ^,^ ^77^ ^,^ ^78^ ^,^ ^85^
Schizophreniform disorder	1 (4%)	^55^
Bipolar disorder	15 (56%)	^55^ ^,^ ^57^ ^,^ ^61^ ^,^ ^62^ ^,^ ^67^ ^,^ ^68^ ^,^ ^72^, ^73^, ^74^, ^75^, ^76^ ^,^ ^79^ ^,^ ^85^, ^86^, ^87^
Depression (with psychosis)	8 (30%)	^55^ ^,^ ^61^ ^,^ ^68^ ^,^ ^72^, ^73^, ^74^ ^,^ ^85^ ^,^ ^87^
Other NOS	7 (26%)	^66^ ^,^ ^68^ ^,^ ^72^ ^,^ ^74^ ^,^ ^75^ ^,^ ^85^ ^,^ ^87^
Unclear	0 (0%)	None
Not reported	7 (21%)	^54^ ^,^ ^59^ ^,^ ^64^ ^,^ ^71^ ^,^ ^82^, ^83^, ^84^
**Study country**		
USA	14 (41%)	^59^ ^,^ ^60^ ^,^ ^63^ ^,^ ^67^ ^,^ ^69^ ^,^ ^71^, ^72^, ^73^, ^74^, ^75^ ^,^ ^77^ ^,^ ^80^ ^,^ ^83^ ^,^ ^87^
Spain	4 (11%)	^54^ ^,^ ^56^ ^,^ ^62^ ^,^ ^85^
Australia	2 (6%)	^55^ ^,^ ^68^
Italy	2 (6%)	^61^ ^,^ ^64^
Switzerland	2 (6%)	^57^ ^,^ ^86^
UK	2 (6%)	^78^ ^,^ ^82^
Brazil	1 (3%)	^66^
Croatia	1 (3%)	^65^
Germany	1 (3%)	^84^
Japan	1 (3%)	^81^
Korea	1 (3%)	^58^
Sweden	1 (3%)	^76^
Taiwan	1 (3%)	^70^
Netherlands	1 (3%)	^79^
Unclear	0 (0%)	None
Not reported	0 (0%)	None
**Study characteristics**		
**Care‐setting**		
Outpatients/community mental health teams	22 (64%)	^56^ ^,^ ^58^, ^59^, ^60^, ^61^ ^,^ ^63^ ^,^ ^66^ ^,^ ^68^ ^,^ ^69^ ^,^ ^71^, ^72^, ^73^, ^74^, ^75^ ^,^ ^77^ ^,^ ^78^ ^,^ ^81^, ^82^, ^83^ ^,^ ^85^, ^86^, ^87^
Inpatients	3 (9%)	^65^ ^,^ ^70^ ^,^ ^84^
Both	3 (9%)	^54^ ^,^ ^55^
Supportive housing	1 (3%)	^76^
Other	3 (9%)	^57^ ^,^ ^79^ ^,^ ^80^
Unclear	0 (0%)	None
Not reported	3 (9%)	^62^ ^,^ ^64^ ^,^ ^67^
**Weight management type**		
Maintenance	2 (6%)	^54^ ^,^ ^55^
Loss	32 (94%)	^56^, ^57^, ^58^, ^59^, ^60^, ^61^, ^62^, ^63^, ^64^, ^65^, ^66^, ^67^, ^68^, ^69^, ^70^, ^71^, ^72^, ^73^, ^74^, ^75^, ^76^, ^77^, ^78^, ^79^, ^80^, ^81^, ^82^, ^83^, ^84^, ^85^, ^86^, ^87^
<6 months	22 (69%)	^56^, ^57^, ^58^, ^59^, ^60^, ^61^, ^62^, ^63^, ^64^, ^65^, ^66^, ^67^, ^68^, ^69^, ^70^, ^71^ ^,^ ^77^ ^,^ ^82^, ^83^, ^84^, ^85^, ^86^, ^87^
7–12 months	10 (31%)	^72^, ^73^, ^74^, ^75^, ^76^ ^,^ ^78^, ^79^, ^80^, ^81^ ^,^ ^83^
Unclear	0 (0%)	None
Not reported	0 (0%)	None
**Facilitator**		
Mental health professionals (e.g., clinical psychologist)	9 (26%)	^54^ ^,^ ^56^ ^,^ ^57^ ^,^ ^66^ ^,^ ^69^ ^,^ ^78^ ^,^ ^79^ ^,^ ^81^ ^,^ ^85^
Other health professional (e.g., nurse)	1 (3%)	^60^
Dietitians	2 (6%)	^55^ ^,^ ^84^
Research staff	6 (18%)	^59^ ^,^ ^67^ ^,^ ^68^ ^,^ ^74^ ^,^ ^82^ ^,^ ^87^
Mix facilitators	3 (9%)	^58^ ^,^ ^86^ ^,^ ^75^
Other (e.g., fitness coaches)	7 (20%)	^62^ ^,^ ^71^, ^72^, ^73^ ^,^ ^76^ ^,^ ^77^ ^,^ ^80^
Unclear	3 (9%)	^64^ ^,^ ^70^ ^,^ ^83^
Not reported	3 (9%)	^61^ ^,^ ^63^ ^,^ ^65^
**Comparison**		
Treatment as usual (TAU)	3 (9%)	^59^ ^,^ ^72^ ^,^ ^73^
Minimal intervention	31 (91%)	^54^, ^55^, ^56^, ^57^, ^58^ ^,^ ^60^, ^61^, ^62^, ^63^, ^64^, ^65^, ^66^, ^67^, ^68^, ^69^, ^70^, ^71^ ^,^ ^74^, ^75^, ^76^, ^77^, ^78^, ^79^, ^80^, ^81^, ^82^, ^83^, ^84^, ^85^, ^86^, ^87^
No intervention	0 (0%)	None
Unclear	0 (0%)	None
Not reported	0 (0%)	None
**Delivery format**		
Individual	13 (38%)	^54^ ^,^ ^55^ ^,^ ^59^ ^,^ ^67^ ^,^ ^70^ ^,^ ^71^, ^72^, ^73^ ^,^ ^77^ ^,^ ^79^ ^,^ ^80^, ^81^, ^82^
Group	16 (47%)	^56^ ^,^ ^57^ ^,^ ^60^, ^61^, ^62^, ^63^ ^,^ ^65^ ^,^ ^66^ ^,^ ^68^ ^,^ ^69^ ^,^ ^75^ ^,^ ^76^ ^,^ ^78^ ^,^ ^84^, ^85^, ^86^
Both	3 (9%)	^58^ ^,^ ^74^ ^,^ ^87^
Unclear	2 (6%)	^64^ ^,^ ^83^
Not reported	0 (0%)	None
**Delivery mode**		
Face‐to‐face	26 (76%)	^54^, ^55^, ^56^ ^,^ ^57^ ^,^ ^60^, ^61^, ^62^, ^63^ ^,^ ^65^, ^66^, ^67^, ^68^, ^69^, ^70^ ^,^ ^72^, ^73^, ^74^ ^,^ ^76^ ^,^ ^77^ ^,^ ^79^, ^80^, ^81^, ^82^ ^,^ ^84^, ^85^, ^86^
Online	1 (3%)	^71^
Other	0 (0%)	None
Mix modes (e.g., face‐to‐face and telephone calls)	5 (15%)	^58^ ^,^ ^59^ ^,^ ^75^ ^,^ ^78^ ^,^ ^87^
Unclear	2 (6%)	^64^ ^,^ ^83^
Not reported	0 (0%)	None
**Outcome**		
↔ no difference in weight loss	20 (59%)	^56^ ^,^ ^57^ ^,^ ^59^ ^,^ ^65^, ^66^, ^67^, ^68^ ^,^ ^70^, ^71^, ^72^ ^,^ ^76^, ^77^, ^78^, ^79^, ^80^ ^,^ ^83^, ^84^, ^85^, ^86^, ^87^
+ outcome change in desired direction (i.e., weight loss)	12 (35%)	^54^ ^,^ ^55^ ^,^ ^58^ ^,^ ^60^, ^61^, ^62^, ^63^ ^,^ ^73^, ^74^, ^75^ ^,^ ^81^ ^,^ ^82^
− outcome change in undesired direction (i.e., weight gain)	0 (0%)	None
Unclear	1 (3%)	^64^
Not reported	1 (3%)	^69^
**Risk of bias score**		
Low	1 (3%)	^80^
High	16 (47%)	^54^ ^,^ ^55^ ^,^ ^58^ ^,^ ^64^, ^65^, ^66^, ^67^, ^68^, ^69^ ^,^ ^71^ ^,^ ^74^ ^,^ ^75^ ^,^ ^78^ ^,^ ^81^, ^82^, ^83^
Unclear	17 (50%)	^56^ ^,^ ^57^ ^,^ ^59^, ^60^, ^61^, ^62^, ^63^ ^,^ ^70^ ^,^ ^72^ ^,^ ^73^ ^,^ ^76^ ^,^ ^77^ ^,^ ^79^ ^,^ ^84^, ^85^, ^86^, ^87^

Abbreviations: NOS, not otherwise reported; RCT, randomized controlled trial.

#### 3.3.4 Risk of bias

Sixteen studies were judged to be at high risk of bias.^54^, ^55^, ^58^, ^64^, ^65^, ^66^, ^67^, ^68^, ^69^, ^71^, ^74^, ^75^, ^78^, ^81^, ^82^, ^83^ One study was judged to be low risk of bias overall.^80^ The remaining 17 studies were rated as unclear risk of bias.^56^, ^57^, ^59^, ^60^, ^61^, ^62^, ^63^, ^70^, ^72^, ^73^, ^76^, ^77^, ^79^, ^84^, ^85^, ^86^, ^87^ Table 2 lists summary risk of bias scores. Appendix H lists judgments by domain for each study.

#### 3.3.5 Qualitative comparative analysis

The results from the exploratory CsQCA are presented in Table 3 and Appendix I. The characteristic with most support for effectiveness was supporting tools, which meant prompts like pedometers and cookery books. The consistency was 0.60 implying that in 60% of interventions that supporting tools were used, the intervention was shown to be effective. The coverage was 0.42, meaning that 42% of effective interventions included this characteristic. Interim booster support was linked with a significant difference in weight loss in favor of the intervention compared with the control 60% of the time and was included in 21% of the effective interventions. Tailored materials achieved a consistency rating of 58% and coverage of 50%.

**TABLE 3:** Results from CsQCA: Intervention characteristics and configurations associated with statistically significant changes in weight loss

	Consistency^a^	Coverage^a^
Characteristics		
Education on specific contributors to weight gain	0.50	0.42
Emphasis on successes and achievements	0.35	0.50
Knowledgeable facilitator	0.37	0.64
Peer support	0.36	0.28
Interim booster support	0.60	0.21
Supporting tools	0.60	0.42
Tailored materials	0.58	0.50
Practical support	0.33	0.14
Incentives	0.33	0.21
Selected configurations of characteristics		
Interim booster support + tailored materials OR interim booster support + knowledgeable facilitator OR interim booster support + supporting tools	0.75	0.21

*Note*: In crisp‐set qualitative comparative analysis (CsQCA), each intervention characteristic scores 1 or 0 to describe whether the intervention did or did not have the characteristic of interest. The outcome in our analysis was whether or not the intervention was associated with statistically significant changes in weight in the desired direction (i.e., weight loss or weight gain prevention). Together these scores form an intervention's configuration, which is the set of conditions associated (=1) or not associated (=0) with statistically significant changes in the outcome.

^a^ Consistency represents the proportion of times interventions were effective when that characteristic was present. Coverage indicates the proportion of interventions that were effective that included this characteristic.

The variety of configurations suggested that no single characteristics or combination of characteristics accounted for all weight loss outcomes. Therefore, we examined patterns among those configurations. Each configuration represents an intervention scenario that is linked to weight loss. An initial examination of these configurations revealed that some configurations appear more consistently than others. The following configurations had the highest consistency and highest coverage: (1) interim booster support plus tailored materials, (2) interim booster support plus a knowledgeable facilitator, (3) interim booster support plus supporting tools. The consistency of these configurations was 0.75 and coverage was 0.21 (see Table 3).

## 4 DISCUSSION

### 4.1 Overview of findings

In the systematic review of qualitative studies, nine characteristics were identified as promoting engagement for people with SMI in weight management interventions. These included the following: (1) education on the specific contributors to weight gain for people with SMI, (2) emphasis on success and achievements, (3) a knowledgeable facilitator, (4) peer support, (5) interim booster support, (6) supporting tools, (7) tailored materials, (8) practical support, and (9) incentives. In the systematic review of RCTs, three of these characteristics were most commonly associated with weight loss. First, interventions that offered supporting tools like pedometers and cookery books. Second, interventions that offered interim booster support between sessions such as low‐intensity telephone calls. Third, interventions that tailored the materials and session structure to account for the impact of a mental health diagnosis—such as low motivation—often faced by people with SMI. There was little evidence that including other intervention characteristics improved effectiveness.

### 4.2 Strengths and limitations

The protocol was published a priori and we used gold standard Cochrane methods, like duplicate screening to minimize bias, with no year or language limits. We included PPI at multiple stages of this review. We also comprehensively reviewed the available data—using both qualitative and systematic methods—to best capture the reality of weight management interventions for people living with SMI. For the exploratory CsQCA, we included only RCTs, which although restricts the nature of studies that our review was able to evaluate, increases confidence in the validity of our results since this design minimizes confounding. The CsQCA is also useful for identifying characteristics that may improve effectiveness and can be used when there are insufficient studies to conduct a component network meta‐analysis. However, CsQCA lacks the ability to isolate the effectiveness of components that a component network meta‐analysis affords.

On limitations, our systematic review of qualitative studies only included 20 studies. This might reflect the lack of available literature or our search strategy since we did not include service evaluations. Furthermore, the approach we took depends upon participants in BWMIs being able to identify characteristics that help promote engagement. Some characteristics that may have assisted engagement in BWMIs may be unapparent and therefore not reported, meaning we could not include them in our CsQCA. Hence, these particular findings ought to be considered preliminary with further confirmatory research required. Moreover, the risk for an SMI diagnosis is higher in ethnic minority groups including Black African, Black Caribbean, South Asian, and mixed ethnicity than White ethnic groups.^88^ There are also ethnic inequalities in the rates of disengagement from health services and physical health outcomes.^89^ Yet, only 55% of the 20 included qualitative studies in our study reported ethnicity and 53% were White. Therefore, the characteristics that promote engagement in BWMIs for ethnic minority groups might not have been captured in our review.

On the systematic review of RCTs, the interventions themselves were incompletely described in most studies, which we attempted to overcome by checking supplementary materials, trial protocols, and contacting authors for more information. Thus, interventions may have included intervention characteristics but not reported it and this lends itself to non‐differential misclassification in our CsQCA. Similarly, omissions in study reporting of RCTs meant assessments of published articles were difficult. This meant we classified most studies as having an unclear risk of bias and the potential for bias reduces the validity of the results. Also, some studies were underpowered so interventions that we declared ineffective may have been effective but the study failed to detect this. This would have reduced the consistency statistics in our CsQCA.

### 4.3 Comparison with other studies

A previous meta‐analysis including 41 studies on the effectiveness of BWMIs for people with SMI reported an approximate 2 kg greater weight loss in interventions versus no support at follow‐ups ranging from 8 to 52 weeks.^18^ However, there was marked heterogeneity between outcomes, which is what we sought to investigate here. We focused on intervention characteristics that specifically addressed barriers that people with SMI have reported when engaging with BWMIs. The interventions included in this review undoubtedly differed in characteristics that are common to BWMIs for the general population, and variation in the effectiveness between them could be explained by these other generic behavioral characteristics. That said, a previous review that examined these characteristics found little evidence that variation in their inclusion explained variation in effectiveness.^90^

We found some of the most effective characteristics of interventions for people with SMI are no different from what is offered in some BWMIs for the general population. Arguably, interim support may serve as a “buffer” against stress through its effect on increased self‐efficacy, while decreasing feelings of emotional and social isolation.^91^ In people with SMI, regular contact is reported to provide a sense of continuity of care and an opportunity to facilitate a high‐quality therapeutic alliance with a healthcare professional.^92^ This may be important for this group when engaging in any treatment option, not just those related to weight loss.^93^

### 4.4 Implications for future research and practice

The majority of interventions examined here were bespoke BWMIs and some were geared specifically for the needs of people with SMI. However, we know of no countries where these are widely available as part of health service provision. In some cases, the interventions in this review have provided such intensive behavioral support that health economic assessments suggest that they are not cost‐effective.^78^ At the same time, people with SMI continue to experience disproportionately high levels of preventable morbidity and mortality compared with the general population for want of effective weight management support.^8^ In the United States and United Kingdom, national guidelines suggest that anyone with overweight or obesity should be offered weight management support.^12^, ^13^ The United Kingdom does provide widely available and publicly funded BWMIs to back‐up this guideline. Moreover, our systematic review of qualitative studies identified issues that may preclude people with SMI engaging with them. The characteristics we have identified from our CsQCA could easily sit alongside the modestly priced BWMIs that are available.^14^ For example, regular interim support is, by its nature, not integral to mainstream services, while supporting tools could likewise be adjunctive. Our findings may encourage researchers to empirically test interventions that add these elements to support engagement with BWMIs and assess the impact on weight and health outcomes in people with SMI. For instance, the *PR*agmatic *E*xplanatory *C*ontinuum *I*ndicator *S*ummary‐2 (PRECIS‐2) may be a useful framework to consider when designing a pragmatic trial given possible implementation issues as an intervention moves from an RCT to the real world.^94^

However, more exploratory research may first be needed to understand how or why interventions are more or less likely to work for people with SMI including different ethnic groups. This should include novel approaches to evaluation, for example, using ethnographic methods or those recommended under the person‐based approach,^95^, ^96^ which would allow an understanding of the context of users and their views of particular characteristics of an intervention to guide trial development. Similarly adjunctive approaches include realist synthesizes to identify underlying causal mechanisms of behavior change.^97^

## 5 CONCLUSIONS

Here we found evidence to suggest people with SMI are more likely to lose weight when offered interventions that provide additional contact between sessions, tools to support enactment, or tailored materials. Mainstream behavioral weight loss interventions that include these features could improve health outcomes for people with SMI but would need to be tested in future trials.

## CONFLICT OF INTEREST

The authors declare no conflict of interest. The views expressed in this publication are those of the author(s) and not necessarily those of the funders. No funders had a role in the study design, data collection, analysis, or interpretation. The research was conducted independently of the funders.

## AUTHOR CONTRIBUTIONS

CL, CP, PA, and FW conceived and participated in the design of the study. CL coordinated the review. CL, CS, MM, AH, and RE undertook the review. CL performed all analyses, wrote the paper, and had primary responsibility for the final content. All authors interpreted the data, read, edited, and approved the final manuscript. CL is the study guarantor.

## Data Availability

The data that support the findings of this study are available from the corresponding author (CL) upon reasonable request.

## APPENDIX A SEMISTRUCTURED TOPIC GUIDE FOR THE PATIENT AND PUBLIC INVOLVEMENT CONSULTATION

**Table:**

Question topic	Researcher question
History	“In what ways did your weight change after your diagnosis?”
Influences	“What do you think contributed to your weight change?”
Attitudes	“Did you do anything to change your weight?”
Challenges	“Were there, if any, challenges to losing weight?”
Current Thoughts	“How do you feel about your weight now?”
Recruitment	“What do you think of group‐based weight management programmes like Weight Watchers or Slimming World?”
Attending Sessions	“Is there anything that would affect your decision to attend?”
Additional Support	“Is there anything else we can provide *in addition* to the programme?”
Peer Support	“What are your thoughts on going with another person?”
Incentivize	“Do you think we can offer people anything to help them to attend programme sessions?”
Other Suggestions	“How else can your healthcare team support your attendance?”
Final Comments	“Are there any final comments or suggestions?”

## APPENDIX B MEDLINE SEARCH STRATEGY FOR THE SYSTEMATIC REVIEW OF QUALITATIVE STUDIES

**Table:**

Search	Search terms
1	serious mental illness.ti,ab
2	weight.ti,ab OR diet. ti,ab OR nutrition. ti,ab
3	qualitative.ti,ab
4	1 and 2 and 3

*Note*: Article search date: 23.09.2020; articles retrieved: *n* = 53.

## APPENDIX C MEDLINE SEARCH STRATEGY FOR THE SYSTEMATIC REVIEW OF RANDOMIZED TRIALS

**Table:**

Search	Search terms
1	“Schizophrenia Spectrum and Other Psychotic Disorders” [Mesh]
2	“Depressive Disorder, Major” [Mesh]
3	“Psychotropic Drugs” [Mesh:NoExp]
4	“Antipsychotic Agents” [Mesh]
5	severe mental illness.ti,ab. OR severely mentally ill.ti,ab. OR serious mental illness.ti,ab. OR severe mental disorder*.ti,ab. OR serious mental disorder*.ti,ab. OR anti‐psychotic*.ti,ab. OR antipsychotic*.ti,ab. OR psychotropic*.ti,ab. OR psycho‐tropic*.ti,ab. OR psychoactive.ti,ab. OR psycho‐active.ti,ab. OR schizophren*.ti,ab. OR psychotic*.ti,ab. OR psychosis.ti,ab. OR delusion*.ti,ab. OR hallucination*.ti,ab. OR disordered speech.ti,ab. OR paranoia.ti,ab. OR major depress*.ti,ab.
6	1 or 2 or 3 or 4 or 5
7	“Obesity” [Mesh]
8	“Body Mass Index” [Mesh]
9	“Body Weight” [Mesh]
10	obes*.ti,ab. OR overweight.ti,ab. OR body weight.ti,ab. OR weight loss.ti,ab. OR weight management.ti,ab. OR weight gain.ti,ab. OR weight change.ti,ab. OR weight reduction.ti,ab. OR weight control.ti,ab. OR body mass.ti,ab. OR bmi.ti,ab.
11	7 or 8 or 9 or 10
12	“Diet, Reducing” [Mesh]
13	“Exercise” [Mesh]
14	diet*.ti,ab. OR nutrition*.ti,ab. OR weight.ti,ab. OR lifestyle.ti,ab. OR exercise.ti,ab. OR physical exercise.ti,ab. OR physical activity.ti,ab.
15	12 or 13 or 14
16	“Healthy Lifestyle” [Mesh]
17	“Weight Reduction Programs” [Mesh]
18	“Health Education” [Mesh:NoExp]
19	“Health Promotion” [Mesh]
20	intervention*.ti,ab OR program*.ti,ab OR education.ti,ab OR promotion.ti,ab OR training.ti,ab OR workshop*.ti,ab
21	16 or 17 or 18 or 19 or 20
22	6 and 11 and 15 and 21
23	randomized controlled trial.pt
24	controlled clinical trial.pt
25	randomized.ti,ab.
26	placebo.ti,ab.
27	randomly.ti,ab.
28	trial.ti,ab.
29	groups.ti,ab
31	drug therapy.fs
31	23 or 24 or 25 or 26 or 27 or 28 or 29 or 30
32	22 and 31

*Note*: Article search date: 11.06.2020; articles retrieved: *n* = 869.

## APPENDIX E PARTICIPANT‐ AND STUDY‐LEVEL CHARACTERISTICS FOR THE SYSTEMATIC REVIEW OF QUALITATIVE STUDIES

**Table:**

Reference	Country	Care‐setting	Recruitment from trial	Population	Participants interviewed (*N*)	Age in years, *m* (SD)	Sex, *n* (%) male	Ethnicity, *n* (%) white	Facilitator	Modality
Aschbrenner et al.^34^	USA	Three public mental health centers	Yes	Diagnosis of schizophrenia, schizoaffective disorder, major depression, or bipolar disorder	30	NR	15 (50%)	NR	One facilitator plus one operator	Six semistructured focus groups each of 3–8 persons
Aschbrenner et al.^35^	USA	A community mental health team	Trial development	Diagnosis of schizophrenia, schizoaffective disorder, bipolar disorder, major depression	10	46.6 (8.7)	1 (10%)	9 (90%)	NR	Semistructured interview; 45–60 min each
Barre et al.^46^	USA	Mental health center	No	SMI NOS	31	Range: 30–61	NR	NR	NR	Semistructured, 1:1 interview
Bochicchio et al.^47^	USA	Supportive housing	Yes	Intervention participants self‐reported with SMI, plus intervention peer specialists and supervisors	Intervention participants: 28 Peer specialists: 4 Supervisors: 5	Intervention participants:49 (9.27) Peer specialists: 44.72 (7.41) Supervisors: 34.25 (10.2)	Intervention participants: 14 (50%) Peer specialists: 2 (50%) Supervisors: 1 (20%)	Intervention participants: 6 (21%) Peer specialists: 1 (25%) Supervisors: 3 (60%)	Trained research assistant	A 1:1 interview; 1 h each
Carey et al.^48^	UK	Community mental health teams	Trial development	A clinical diagnosis of schizophrenia, schizoaffective disorder or FEP	Unclear	NR	NR	NR	NR	Participants were invited to one of four pilot cohorts to provide feedback
Every‐Palmer et al.^49^	New Zealand	Three medium secure units; one minimum secure; and one unlocked unit	No	ICD diagnosis of schizophrenia, schizoaffective disorder, bipolar disorder, or psychosis NOS	51	38 (10.4)	40 (78%)	0 (0%)	Researcher known to the participant	Semistructured, 1:1 interview; 40 min each
Gandhi et al.^50^	South India	Tertiary mental health institute	No	ICD‐10 diagnosis of schizophrenia spectrum disorders	5 + 13 caregivers	43.2 (NR)	NR	NR	Researcher NOS	Semistructured interview; 40–60 min each
Gossage‐Worral et al.^51^	UK	Ten English NHS mental health trusts in urban and rural locations	Trial process evaluation	A clinical diagnosis of schizophrenia, schizoaffective disorder or FEP (defined as <3 years since presentation to mental health services)	Intervention participants: 24	Range: 18–55	12 (50%)	20 (83.3)	NR	Semistructured telephone interview; median duration: 18.87; range: 13.06 to 30.33 min
Jimenez et al.^52^	USA	NR	Yes	DSM‐IV diagnosis of schizophrenia, schizoaffective disorder, bipolar disorder, or major depression	20	40.25 (10.4)	11 (55%)	0 (0%)—all participants Latino	NR	Semistructured, 1:1 interview; 60–90 min each
Lesley et al.^53^	USA	NR	Yes	SMI NOS	11	NR	2 (18%)	NR	Nurse researcher	A 1:1 interview
Muralidharan et al.^36^	USA	Greater Los Angeles Veterans Affairs Medical Centre	Yes	Schizophrenia spectrum disorders, affective psychoses, or posttraumatic stress disorder	48	NR	NR	NR	NR	NR
Nover^37^	USA	A community clinic	Yes	SMI NOS	11	Range: 45–63	2 (18%)	11 (100%)	Lead author	Semistructured, 1:1 interview; 45 min each
O'Hara et al.^38^	USA	Supportive housing	Trial development	Self‐reported SMI including schizophrenia or schizoaffective disorder, bipolar disorder, major depression	8	NR for the qualitative study	NR for the qualitative study	NR for the qualitative study	Research assistant	Two focus groups and field notes
Olmos‐Ochoa et al.^39^	USA	NR	Yes	DSM‐IV diagnosis of schizophrenia, schizoaffective disorder, bipolar disorder, recurrent major depressive disorder with psychosis, or chronic posttraumatic stress disorder	Participants from MOVE! SMI: 24 Participants from WebMOVE: 24	Participants from MOVE! SMI: 53.7 (10.5) Participants from WebMOVE: 45.4 (6.0)	Participants from MOVE! SMI: Unclear (21%) Participants from WebMOVE: Unclear (19%)	Participants from MOVE! SMI: 12 (50%) Participants from WebMOVE: 7 (29.2%)	Three assessors NOS	A 1:1 interview; 15–30 min
Park et al.^40^	Australia	NR	Yes	Diagnosed with schizophrenia	10	Range: 30–65	2 (20%)	NR	Mental health professional not involved in the RCT	Interview in a setting of participants' choice; 30–60 min
Pearsall et al.^41^	UK	A community mental health team	No—perspectives of those who declined to participate in a trial	Diagnosis of schizophrenia, schizoaffective or bipolar affective disorder	13	54.6 (NR)	Unclear (50%)	NR	Lead author	Interview at the community base or participants' home; 30–40 min
Sayer et al.^42^	USA	A supportive housing building and nearby neighbourhoods	No	SMI NOS	55. Note: only 38 participants provided personal and demographic characteristics	52.4 (NR)	17 (44.7%)	0 (0%)—all participants were African American	Team leader with experience of mental illness	Five focus groups with 6–12 persons; 90 min each
Shiner et al.^43^	USA	NR	Yes	SMI NOS	8	43.0 (±15.3)	5 (62.5%)	8 (100%)	Lead author	Semistructured, 1:1 interview; 1 h each
Vazin et al.^44^	USA	Six psychiatric rehabilitation program sites	Yes	Diagnosis of schizophrenia, schizoaffective disorder, bipolar disorder, major depression or other diagnosis	20	Range: 20–70	10 (50%)	14 (70%)	Intervention staff	Semistructured, 1:1 interview; 20–30 min each
Yarborough et al.^45^	USA	Three community mental health clinics	Yes	Diagnosis of schizophrenia or schizoaffective disorder, bipolar disorder, affective psychosis, or PTSD	84	48.1 (10.1)	30 (36%)	66 (79%)	Master's and doctoral level research staff	A 1:1 interview

*Note*: DSM‐IV, Diagnostic and Statistical Manual of Mental Health Disorders, 4th Edition; FEP, first episode psychosis; ICD‐10, International Statistical Classification of Diseases and Related Health Problems 10th Edition; NOS, not otherwise specified; NR, not reported; SMI, serious mental illness; 1:1, one‐to‐one.

## APPENDIX F PARTICIPANT‐LEVEL CHARACTERISTICS FOR THE SYSTEMATIC REVIEW OF RANDOMIZED TRIALS

**Table:**

Reference	Country	Care‐setting	Recruitment	Population	Participants randomized (*N*)	Age, *m* (SD)	Sex, *n* (%) male	Ethnicity, *n* (%) white
**Prevention—First episode psychosis**								
Álvarez‐Jiménez et al.^54^	Spain	Outpatients and inpatients	Referral from primary care services, emergency services, and mental health professionals	DSM‐IV criteria for schizophrenia, schizophreniform disorder, schizo‐affective disorder, delusional disorder, brief reactive psychosis or psychosis NOS	61	26.8 (7.7)	46 (75.4%)	NR
Evans et al.^55^	Australia	Community and inpatients	NR	NR. Both first‐episode and previously diagnosed subjects were included. Sub‐group analyzes by diagnosis duration not reported.	51	NR	22 (43.1%)	NR
**Stabilized psychosis—intervention** ***≤*** **6 months**								
Attux et al.^66^	Brazil	Outpatients	Referral from a clinician or a mental health worker	DSM‐IV diagnosis of schizophrenia spectrum	160	NR	96 (60.0%)	118 (73.8%)
Brar et al.^77^	USA	Outpatients from 19 sites in the USA	From a prior study conducted by the authors	DSM‐IV diagnosis of schizophrenia or schizoaffective disorder	72	NR	29 (40.2%)	35 (48.6%)
Brown et al.^82^	UK	Community mental health team	Advertised by posters and key workers to people on the caseload	ICD‐10 primary diagnosis of psychosis, major affective illness or severe personality disorder	28	NR	4 (14.3%)	NR
Cordes et al.^84^	Germany	Inpatients at the Department of Psychiatry and Psychotherapist, Heinrich Heine University	Inpatients were assessed for eligibility and then agreed to participate	DSM‐IV criteria for schizophrenia or schizoaffective disorder (according to the Mini International Neuropsychiatric Interview)	74	NR	42 (56.7%)	NR
Fernandez Guijarro et al.^85^	Spain	Community mental health centers	Participants were recruited from a previous cross‐sectional study	SMI NOS	61	46.9 (9.1)	41 (67.2%)	NR
Gillhoff et al.^86^	Switzerland	Outpatients of a psychiatric hospital, associated psychiatrists, and advertisement in local newspapers	NR	Self‐reported bipolar confirmed with the Mini International Neuropsychiatric Interview	50	48 (range 20–65 years)	27 (54.0%)	NR
Goldberg et al.^87^	USA	Veteran outpatient mental health clinics	NR	DSM‐IV diagnosis of schizophrenia, other psychotic spectrum disorder, bipolar disorder, major depression, or severe anxiety disorder	109	52.0 (69.1)	88 (81.0%)	36 (68.0%) African American
Iglesias‐Garcia et al.^56^	Spain	Outpatients attending a community mental health center	NR	DSM‐IV diagnosis of schizophrenia	15	39.9 (11.3)	11 (73.3%)	11 (68.8%)
Khazaal et al.^57^	Switzerland	Participants were recruited from the University Department of Adult Psychiatry and through referral by local mental health providers affiliated with the department	NR	SMI NOS	61	40.7 (10.3)	28 (45.9%)	NR
Kwon et al.^58^	Korea	Outpatients across 4 clinical centers	NR	DSM‐IV diagnosis of schizophrenia or schizoaffective disorder	48	NR	15 (31.2%)	NR
Lee et al.^59^	USA	Outpatients from community mental health centers	NR	SMI incl. Schizophrenia, schizoaffective disorders, bipolar disorders, and major depressive disorder	19	Median age (IQR due to low sample size): 57 (48–62)	7 (36.8%)	15 (79.0%)
Littrell et al.^60^	USA	Community mental health centers and private practice psychiatrists	Referrals	DSM‐IV diagnosis of schizophrenia or schizoaffective disorder	70	NR	43 (61.4%)	52 (74.3%)
Mauri et al.^61^	Italy	Outpatients	NR	NR	45	38.9 (range: 19–60)	14 (42.4%)	NR
Masa‐Font et al.^62^	Spain	NR	NR	Diagnosis of schizophrenia, schizoaffective or bipolar disorder	332	NR	182 (54.8%)	NR
McKibbin et al.^63^	USA	Board‐and‐care facilities, day treatment programs and community clubhouses	NR	Physician‐confirmed diagnoses of schizophrenia and type II diabetes	64	NR	37 (57.8%)	35 (54.6%)
Milano et al.^64^	Italy	NR	NR	DSM‐IV diagnosis of schizophrenia	NR	NRA:	16 (44.4%)	NR
Soric et al.^65^	Croatia	Inpatients in a psychiatric hospital	Word of mouth	Schizophrenia NOS	79	NR	57 (72.0%)	NR
Sylvia et al. ^67^	USA	NR	NR	Primary diagnosis of bipolar disorder	38	42.0 (12.3)	32 (84.2%)	32 (84.2%)
Usher et al.^68^	Australia	Five local mental health services including NGOs	Posters displayed at local community mental health services, NGOs, word of mouth	Self‐reported SMI including schizophrenia, bipolar disorder, and other psychotic disorders	101	NR	54 (53.5%)	72 (71.3%)
Weber et al.^69^	USA	Mental health clinics	The PI used flyers in the clinic as well as working with the case managers and medical	DSM IV‐TR (DSM) criteria for schizophrenia or schizoaffective disorder	17	NR	5 (29.4%)	5 (29.4%)
Wu et al.^70^	Taiwan	Inpatients	NR	DSM‐IV diagnosis of schizophrenia	53	NR	22 (42.0%)	NR
Young et al.^71^	USA	Mental health clinics	For recruitment, we obtained a list of patients who met inclusion criteria for psychiatric diagnosis, age, and psychotropic medication. Study flyers were also posted in mental health clinics	Diagnosis of schizophrenia, schizoaffective disorder, bipolar disorder, major depressive disorder with psychosis, or posttraumatic stress disorder.	276	NR	226 (81.8%)	94 (34.1%)
**Stabilized psychosis—intervention 7–12 months**								
Bartels et al.^72^	USA	A community mental health center in Concord	NR	DSM–IV diagnosis of schizophrenia, schizoaffective disorder, bipolar disorder, major depression (based on the Structured Clinical Interview)	133	43.8 (11.5)	51 (38.0%)	122 (92.0%)
Bartels et al.^73^	USA	Three community mental health providers	NR	DSM–IV diagnosis of schizophrenia, schizoaffective disorder, bipolar disorder, major depression (based on the Structured Clinical Interview)	210	43.9 (11.2)	103 (49.0%)	113 (54.0%)
Brown et al.^64^	USA	Community mental health programs	NR	SMI NOS	136	NR	45 (33.1%)	81 (59.6%)
Daumit et al.^74^	USA	Community psychiatric rehabilitation programs or their outpatient mental health clinic	Study staff recruited participants by means of presentations at study sites and received referrals from rehabilitation program staff	SMI NOS. Minimal inclusion criteria enroll a broad population that would be representative of persons with SMIs.	291	45.3 (11.3)	145 (49.8%)	163 (56.0%)
Green et al.^75^	USA	Community mental health centers	Electronic medical records and clinician referral	NR	200	47.2 (10.6)	56 (28.0%)	174 (87.7%)
Forsberg et al.^76^	Sweden	Persons with a psychiatric disability and their staff working with housing support or in supported housing facilities	NR	DSM‐IV diagnosis of schizophrenia, bipolar disorder, personality disorders, other psychotic disorders and autism spectrum disorders with no or mild cognitive impairments	49	NR	25 (61.0%)	NR
Holt et al.^78^	UK	Ten English NHS mental health trusts in urban and rural locations	From clinic lists and case notes. Posters and leaflets encouraged self‐referral.	A clinical diagnosis of schizophrenia, schizo‐affective disorder or FEP (defined as <3 years since presentation to mental health services)	412	NR	210 (50.9%)	349 (84.7%)
Looijmans et al.^79^	Netherlands	Mental health organizations	Invitation by mental health nurse at annual review	SMI NOS	284	46.1 (10.8)	120 (49.2%)	NR
Lovell et al.^80^	USA	Early intervention services	Case notes of service users were screen and potentially eligible participants were contacted by the researcher	Diagnosis of schizophrenia, schizophreniform disorder, schizoaffective disorder, delusional disorder, brief reactive psychosis, or psychosis NOS; FEP occurring within the 3 years preceding the trial	105	25.7 (5.7)	63 (60.0%)	86 (82.0%)
Sugawara et al.^81^	Japan	Outpatient settings	NR	Diagnosis of schizophrenia according to DSM‐IV or ICD‐10	265	NR	98 (51.9%)	NR

*Note*: BMI, body mass index; ICD‐10, International Statistical Classification of Diseases and Related Health Problems 10th Edition; ICQ, interquartile range; DSM‐IV, Diagnostic and Statistical Manual of Mental Health Disorders, 4th Edition; FEP, first episode psychosis; NOS, not otherwise specified; NR, not reported; SMI, serious mental illness; WC, waist circumference.

## APPENDIX G STUDY‐LEVEL CHARACTERISTICS FOR THE SYSTEMATIC REVIEW OF RANDOMIZED TRIALS

**Table:**

Reference	Theoretical basis of intervention	Delivery format and mode	Facilitator	Sessions offered	Comparison	Outcomes assessed	Outcome results	Between‐group difference at time point	*p* value
**Prevention—First episode psychosis**									
Álvarez‐Jiménez et al.^54^	NR	Individual, face‐to‐face	Clinical psychologists	1–14 sessions NOS	TAU	Weight & BMI	+	*t* = −2.62, *df* = 59	*p* < 0.1
Evans et al.^55^	NR	Individual, face‐to‐face	Dietitians	6 sessions for 60 min	TAU + booklet	Weight, BMI, WC	+	NR	*p* = 0.002
**Stabilized psychosis**—**intervention** ***≤*** **6 months**									
Attux et al.^66^	NR	Group, face‐to‐face	Mental health professionals i.e., nurses, occupational therapists, psychologists and dietitians	12 sessions NOS	TAU	Absolute weight change & BMI	↔	NR	*p* = 0.093
Brar et al.^77^	NR	Individual, face‐to‐face	Group leader NOS	20 sessions incl. 2 therapy sessions per week for 6 weeks followed by 1 session per week for 8 weeks	TAU	Mean weight change	↔	NR	ITT analysis: *p* = 0.120; completers only: *p* = 0.76
Brown et al.^82^	NR	Individual, face‐to‐face	Research staff NOS	6 sessions 1 per week for 50 min	TAU + health promotion package at the end of the intervention	Mean weight change and BMI	+	Mann–Whitney U test: 47.5	*p* = 0.01
Cordes et al.^84^	NR	Group, face‐to‐face	A dietitian experienced in counseling patients with schizophrenia	12 session 1 bi‐weekly for 90 min	TAU	Absolute weight change, BMI, WC	↔	NR	*p* = 0.597
Fernandez Guijarro et al.^85^	NR	Group, face‐to‐face	Mental health nurses	24 sessions NOS	TAU	Mets criteria, which included absolute weight change, BMI and WC	↔	NR. Mann–Whitney‐U‐test.	*p* = 0.919
Gillhoff et al.^86^	NR	Group, face‐to‐face	Psychotherapist, psychiatrist, and fitness trainers	12 sessions	WLC	Absolute weight, BMI, WC	↔	NR	*p* = 0.08
Goldberg et al.^87^	NR. The intervention was adapted for people with SMI to include psychoeducation focusing on nutritional counseling, caloric expenditure, and portion control. The authors also emphasized behavioral and motivational self‐management strategies	Individual and group face‐to‐face with phone calls	Research staff with previous experience in psychosocial and behavioral interventions and with seriously mentally ill adults	Months (1–4 inclusive): weekly. Months (5–6 inclusive): fortnightly	TAU + monthly weigh‐ins and handouts	Absolute weight change, BMI, WC	↔	*F* = 0.13, *df* 1 and 84	*p* = 0.720
Iglesias‐Garcia et al.^56^	NR	Group, face‐to‐face	Accredited psychiatric nurse	12 sessions 1 per week for 60 min over 3 months	The control group attended the clinic once a week, only to assess the anthropometric parameters	Absolute weight change, BMI, WC	↔	NR	*p* = 0.7
Khazaal et al.^57^	CBT NOS	Group, face‐to‐face	Psychologists with master's level training and 2 years of clinical experience in CBT	12 sessions 1 weekly for 2 h	Brief nutritional education	Absolute weight and BMI	↔	NR	NR
Kwon et al.^58^	CBT NOS	Individual and group face‐to‐face with phone calls	Dietitian & exercise coordinator	8 sessions delivered over 12 weeks; once per week for 4 weeks, then once every other week up until week 12	TAU	% weight change, BMI	+	NR	Unclear
Lee et al.^59^	NR	Individual, face‐to‐face with phone calls	Lead author who was a psychiatric nurse practitioner	1 call per week; 1 in‐person session per month	TAU + monthly newsletters	Median (IQR) BMI and WC	↔	NR	NR
Littrell et al.^60^	NR	Group, face‐to‐face	Nurse practitioner/clinician	16 sessions 1 per week for 60 min	NR	Absolute weight change, BMI	+	*t* = 2.93, *df* = 68	*p* = 0.005
Mauri et al.^3^, ^61^	NR	Group, face‐to‐face	NR	NR	Control NOS	Mean change in weight and BMI	+	NR	*p* < 0.01
Masa‐Font et al.^62^	NR	Group, face‐to‐face	Group leaders NOS	NR	TAU	Absolute BMI change	+	NR	*p* = 0.038
McKibbin et al.^63^	Social cognitive theory	Group, face‐to‐face	NR	24 session 1 per week for 90 min	TAU + brochures	Absolute weight, BMI and WC	+	Mixed‐model analysis of variance (ANOVA): *df* = 1,54; *F* = 15.0	*p* < 0.001
Milano et al.^64^	NA. Calorie restriction	Unclear	Unclear	NA	TAU	Absolute weight change, BMI	Unclear	NR	*p* < 0.005
Soric et al.^65^	NR	Group, face‐to‐face	NR	4 sessions NOS	TAU. The control group continued to follow the standard hospital diet and participated in the same nutrition education program as the intervention group.	Absolute weight change, BMI, WC	↔	NR	*p* = 0.943
Sylvia et al.^67^	CBT NOS	Individual, face‐to‐face	Study clinicians incl. Therapists (i.e., Masters‐level students in psychology doctoral programs)	18 sessions over 20 weeks	TAU + WLC	Absolute weight, BMI and WC	↔	NR	*p* > 0.05
Usher et al.^68^	Primary health promotion + motivational interviewing	Group, face‐to‐face	Research staff incl. Mental health nurses		TAU + booklet	Absolute weight and BMI	↔	Unpaired *t*‐test: 0.891	*p* = 0.420
Weber et al.^69^	CBT NOS	Group, face‐to‐face	Psychiatric nurse practitioner		TAU	Mean change in weight and BMI	NR. There were no (within‐group) significant differences in weight, WHR, or BMI scores pretest and posttest based on *t*‐test results		
Wu et al.^70^	NA. Calorie restriction	Individual, face‐to‐face	Unclear	NA	NR	Mean weight change, BMI, WC	↔	NR	NR. Weight and BMI at 3 and 6 months were not significantly lower within the groups nor was there a difference between the control and study groups
Young et al.^71^	NR	Individual, online	A peer wellness coach	Weekly for 6 months	TAU + brochure	Weight and BMI	↔	*F* = 0.91	*p* = 0.40
**Stabilized psychosis**—**intervention 7–12 months**									
Bartels et al.^72^	NR	Individual, face‐to‐face meetings with a fitness coach and dietitian	Health mentor	Once a week for 45–60 min at a fitness club which included fitness coaching and discussion about nutrition + individual meetings with a dietitian for group cooking classes and grocery store tours	The comparison condition also consisted of a free membership to the same local fitness club and included an introduction to the exercise equipment and educational materials on the health benefits of exercise and healthy diet	Absolute weight change, BMI	*↔*	Main effect calculated for 3–12 months was adjusted for baseline value as a covariate. ES (calculated at end point not overall group effect): 0.00, *df*(1,120), *F* = 0.03	*p* = 0.858
Bartels et al.^73^							+	Main effect calculated for 3–12 months was adjusted for baseline value as a covariate: *df* = 1,185; *F* = 4.9;	*p* = 0.029
Brown et al.^83^	NR	Unclear	Unclear	Intensive phase (weeks 1–12): weekly 3‐h sessions. Maintenance phase (weeks 13–24): once a month for 3 h and weekly phone calls. Intermittent supports (weeks 25–52): weekly phone calls and monthly mailings with tips, reminders and praise	TAU	Absolute and mean weight change	↔	The mixed model analysis indicated a significant difference between the intervention and control group at 3 months (the end of the intensive phase) (*F* = 6.936, *p* = 0.01) but not at 6 months (*F* = 1.527, *p* = 0.22) or 12 months (*F* = 0.522, *p* = 0.47)	
Daumit et al.^74^	Social cognitive and behavioral self‐management theories	Individual and group face‐to‐face weight‐management sessions; group exercise sessions	Trained members of staff NOS	Intensive phase (month 1–6): Group weight‐management class: once/week for 45 min for 3/4 weeks; individual visit: once per month for 15‐20 min; group physical activity class: once per month for 50 min; weight in: once per week for 2 min. Details during the maintenance phase are reported in the paper.	TAU	Mean weight and BMI	+	A likelihood based mixed‐effects model, with weight as a function of study‐group assignment and study visit (at baseline and at 6, 12, and 18 months) and with missing data treated as missing at random. The model‐based estimates of the mean difference in changes in weight (the change in the intervention group minus the change in the control group) between the two groups at 6, was −1.5 kg (95% CI, −2.6 to −0.4)	*p* = 0.007
Green et al.^75^	NR	Group face‐to‐face meetings with phone calls	Two facilitators; 1 mental health counselor and an unregistered dietitian with training in nutritional interventions	Weekly 2‐h group meetings with 20 min of physical activity, delivered over 6 months	TAU	Mean weight and BMI	+	Co‐efficient (Values represent the coefficient for the time‐by‐group indicators estimated from the generalized estimating equation models): −4.37; 95% CI: −6.96 to −1.78;	*p* = 0.004
Forsberg et al.^76^	NR	Group, face‐to‐face	Fitness instructor with a personal interest in healthy food but no training or experience in mental health	Twice weekly for 2 h for the duration of the 12 month program	Arts and crafts support	Absolute weight and BMI	↔	NR	NR
Holt et al.^78^	MRC framework for complex interventions. The authors considered three areas that are core to weight‐management interventions in people with SMI: (a) behavior change theory specifically with a focus on food and physical activity; (b) psychological processes underlying weight management; (c) challenges of living with psychosis and its impact on eating and weight.	Group, face‐to‐face with telephone calls	Mental health professionals	4 × 2.5 h foundation group education sessions over 4 consecutive weeks; 3 × 2.5 h ‘booster’ sessions at 3‐monthly intervals. Then, fortnightly support by telephone. Then, 1:1 support contact by telephone, lasting about 10 min, approx. every 2 weeks for the rest of the intervention period	TAU	Weight and BMI	↔	NR	*p* = 0.963
Looijmans et al.^79^	NR	Individual, face to face + individual access online web tool	Mental health nurses	NR	TAU	Absolute BMI and WC	↔	*β*: 1.47 [CI: −0.17; 3.11]	*p* = 0.08. Reporting time point unclear
Lovell et al.^80^	Leventhal's Common Sense Model	Individual, face‐to‐face meetings with a fitness coach and dietitian	Recovery workers	7 sessions over 6 months with a booster session at 9–10 months	TAU	Mean weight change, BMI, WC	↔	*t* = −0.5 (*df*: 91)	*p* = 0.65
Sugawara et al.^81^	NR	Individual, face‐to‐face meetings with a fitness coach and dietitian	Psychiatrists. Participants in group C also attended individual nutritional education sessions conducted monthly by qualified dietitians	Unclear for group B. Monthly and split into 3 phases for group C	The participants were randomly assigned to a standard care (A), doctor's weight loss advice (B), or an individual nutritional education group (C)	Absolute weight change, BMI, WC	+	NR	Group A vs Group B: *p* = 0.384 given. Group B vs Group C: *p* = 0.005 given. Group A vs Group C: *p* < 0.001

*Note*: BMI, body mass index; NA, not applicable; NOS, not otherwise specified; NR, not reported; TAU, treatment as usual; WC, waist circumference; WLC, waitlist control; ↔ no difference in outcome (i.e., no change in weight); + outcome change in desired direction (i.e., weight loss); − outcome change in undesired direction (i.e., weight gain); NS, not significant. Outcome results: Time point closest to the intervention completion.

## APPENDIX H RISK OF BIAS JUDGMENTS BY DOMAIN FOR EACH STUDY IN THE SYSTEMATIC REVIEW OF RANDOMIZED TRIALS

**Table:**

Reference	Random sequence generation (selection bias)	Allocation sequence concealment (selection bias)	Blinding of outcome assessment (detection bias)	Incomplete outcome data (attrition bias)	Selective outcome reporting (reporting bias)	Other bias	Overall
Álvarez‐Jiménez et al.^54^	Low	Unclear	High	Low	Unclear	NA	High
Attux et al.^66^	Low	Unclear	Unclear	High	Low	NA	High
Bartels et al.^72^	Unclear	Unclear	Low	Low	Low	NA	Unclear
Bartels et al.^73^	Unclear	Unclear	Low	Low	Low	NA	Unclear
Brar et al.^77^	Unclear	Unclear	Unclear	Low	Unclear	NA	Unclear
Brown et al.^82^	Low	Unclear	Low	High	Unclear	NA	High
Brown et al.^83^	Low	High	Low	Unclear	High	High	High
Cordes et al.^84^	Unclear	Unclear	Unclear	Low	Low	NA	Unclear
Daumit et al.^74^	Unclear	Unclear	Low	Low	High	NA	High
Evans et al.^55^	Unclear	Unclear	Unclear	High	Unclear	High	High
Fernandez Guijarro et al.^85^	Low	Unclear	Low	Low	Low	NA	Unclear
Forsberg et al.^76^	Low	Unclear	Unclear	Low	Unclear	NA	Unclear
Gillhoff et al.^86^	Unclear	Unclear	Unclear	Low	Low	NA	Unclear
Goldberg et al.^87^	Unclear	Unclear	Unclear	Low	Unclear	NA	Unclear
Green et al.^75^	Low	Low	Low	Low	High	NA	High
Holt et al.^78^	Low	High	Low	Low	Low	Unclear	High
Iglesias‐Garcia et al.^56^	Low	Unclear	Low	Low	Unclear	NA	Unclear
Khazaal et al.^57^	Unclear	Unclear	Unclear	Low	Unclear	NA	Unclear
Kwon et al.^58^	Unclear	Unclear	Unclear	High	Unclear	High	High
Lee et al.^59^	Unclear	Unclear	Unclear	Low	Unclear	NA	Unclear
Littrell et al.^60^	Unclear	Unclear	Unclear	Low	Unclear	NA	Unclear
Looijmans et al.^79^	Low	Unclear	Low	Low	Unclear	NA	Unclear
Lovell et al.^80^	Low	Low	Low	Low	Low	NA	Low
Mauri et al.^61^	Unclear	Unclear	Unclear	Low	Unclear	NA	Unclear
Masa‐Font et al.^62^	Unclear	Unclear	Low	Low	Low	NA	Unclear
McKibbin et al.^63^	Unclear	Unclear	Unclear	Low	Unclear	NA	Unclear
Milano et al.^64^	Unclear	Unclear	Unclear	Low	Unclear	High.	High
Soric et al.^65^	Low	Unclear	High	Low	High	NA	High
Sugawara et al.^81^	Unclear	Unclear	Unclear	Low	Low	High	High
Sylvia et al.^67^	Unclear	Unclear	Low	Low	High	NA	High
Usher et al.^68^	Unclear	Low	Low	Low	Unclear	High	High
Weber et al.^69^	Unclear	Unclear	Low	High	Unclear	High	High
Wu et al.^70^	Unclear	Unclear	Unclear	Low	Unclear	NA	Unclear
Young et al.^71^	Unclear	Unclear	Low	High	Unclear	NA	High

It is not possible to blind participants or study personnel to allocation so this domain was removed.

## APPENDIX I DATA MATRIX FOR THE CRISP‐SET QUALITATIVE COMPARATIVE ANALYSIS (CsQCA) OF RANDOMIZED TRIALS

**Table:**

References	Characteristics									Outcome
	Targeted education	Beliefs and self‐efficacy	Supporting tools	Counseling support	Peer support	Interim support	Tailored materials	Practical support	Incentives	Statistically significant (*P ≤* 0.05) between‐group difference in weigh
Álvarez‐Jiménez et al.^54^	1	0	1	1	0	0	0	0	0	1
Attux et al.^66^	0	1	0	1	1	0	0	0	0	0
Bartels et al.^72^	0	1	0	1	0	0	0	1	1	0
Bartels et al.^73^	0	1	0	1	0	0	0	1	1	1
Brar et al.^77^	0	1	1	0	0	0	0	0	0	0
Brown et al.^82^	0	0	0	0	0	0	0	0	0	1
Brown et al.^83^	0	1	0	0	1	1	0	0	0	0
Cordes et al.^84^	1	1	0	1	0	0	1	0	0	1
Daumit et al.^74^	0	1	1	1	1	0	1	0	1	1
Evans et al.^55^	0	0	0	0	0	0	0	0	0	1
Fernandez Guijarro et al.^85^	1	0	1	1	0	0	0	0	0	0
Forsberg et al.^76^	0	1	0	1	1	0	1	0	1	0
Gillhoff et al.^86^	1	1	0	1	0	0	0	1	0	1
Goldberg et al.^87^	1	1	0	1	1	0	1	0	0	0
Green et al.^75^	1	1	1	1	1	1	1	0	0	1
Holt et al.^78^	1	1	1	1	1	1	1	1	1	0
Iglesias‐Garcia et al.^56^	0	0	0	1	0	0	0	0	0	0
Khazaal et al.^57^	1	1	0	1	1	0	0	0	0	0
Kwon et al.^58^	0	0	0	0	0	0	0	0	0	1
Lee et al.^59^	0	0	0	1	0	1	1	0	0	0
Littrell et al.^60^	0	0	1	1	1	1	1	0	0	1
Looijmans et al.^79^	1	1	0	1	0	0	0	0	0	0
Lovell et al.^80^	0	0	0	0	1	0	0	0	0	0
Mauri et al.^61^	1	0	0	0	0	0	1	0	0	1
Masa‐Font et al.^62^	0	0	0	1	0	0	0	0	0	0
McKibbin et al.^63^	0	1	0	0	1	0	1	0	1	1
Milano et al.^64^	0	0	0	0	0	0	0	0	0	0
Soric et al.^65^	0	0	0	0	0	0	0	0	0	0
Sugawara et al.^81^: IG: B	0	0	0	1	0	0	0	0	0	0
Sugawara et al.^81^: IG: C	0	0	1	1	0	0	0	0	0	1
Sylvia et al.^67^	0	1	0	1	0	0	0	0	1	0
Usher et al.^68^	0	1	1	1	0	0	1	1	1	0
Weber et al.^3^, ^69^	0	1	0	1	0	0	0	1	0	0
Wu et al.^70^	0	0	0	0	0	0	0	0	1	0
Young et al.^71^: IG: MOVESMI	1	1	0	1	0	0	1	0	0	0
Young et al.^71^: IG: WebMOVE	1	1	1	1	0	1	1	0	0	1

*Note*: Two studies are included twice in the crisp‐set qualitative comparative analysis (CsQCA) because they each contributed to two intervention arm. IG: intervention group.
